# Clinical Outcomes in Patients With CLL Treated With BTKi at a Large US Cancer Center

**DOI:** 10.1155/ah/7492594

**Published:** 2025-11-30

**Authors:** Kevin H. Lin, Lynn Huynh, Xiaoqin Yang, Enrico Zanardo, Daria Liborski, Mikaela M. McDonough, Mohammed Z. H. Farooqui, Enrico De Nigris, Shravanthi R. Gandra, Mei Sheng Duh, Jennifer R. Brown, Matthew S. Davids

**Affiliations:** ^1^ Department of Medical Oncology, Dana-Farber Cancer Institute, Newton, Massachusetts, USA, dana-farber.org; ^2^ Analysis Group, Inc., Boston, Massachusetts, USA; ^3^ Merck & Co., Inc., Rahway, New Jersey, USA; ^4^ MSD (UK) Limited, London, UK

**Keywords:** Bruton’s tyrosine kinase inhibitor (BTKi), chart review, chronic lymphocytic leukemia (CLL), overall survival, relapsed/refractory

## Abstract

Patients with chronic lymphocytic leukemia (CLL) treated with covalent Bruton’s tyrosine kinase inhibitors (BTKi) eventually discontinue treatment, but data on outcomes post‐BTKi treatment discontinuation are limited. In this single‐institution, retrospective chart review of 104 adult patients with CLL treated with a covalent BTKi between July 2011 and April 2019 and who subsequently discontinued for any reason, the majority of patients (59.9%) received the index BTKi in their first (27.5%) or second (32.4%) line of therapy. Median progression‐free survival (PFS) from index BTKi initiation was 3.2 years, and median overall survival (OS) was 8.9 years. There was a notable correlation between PFS and OS (*r* = 0.79). Following the discontinuation of their last BTKi treatment, over half of these patients received subsequent therapies (80.6% of whom received regimens containing B‐cell lymphoma 2 inhibitors [BCL2i]). Of the patients who received BCL2i‐containing therapy after index BTKi treatment, 77.8% achieved an overall response to BCL2i at first clinical assessment. However, 73.8% of the patients exposed to BTKi and BCL2i eventually discontinued BCL2i treatment and 36.1% died, with a median follow‐up of 2.6 years from BCL2i initiation. A subset of patients who were exposed to and were found to be relapsed, refractory, resistant, or intolerant to both BTKi and BCL2i‐based regimens (*n* = 25) had high rates of progressive disease at first assessment (15.0%) and death (44.0%). These findings highlight an unmet clinical need for patients with CLL and underscore the urgency to develop effective new strategies to treat those patients who have already received covalent BTKi and BCL2i.

## 1. Introduction

The treatment landscape of chronic lymphocytic leukemia (CLL) has been reshaped over the past decade, largely due to the approval of small molecule inhibitors targeting Bruton’s tyrosine kinase (BTK) and the anti‐apoptotic protein B‐cell lymphoma 2 (BCL2). The first‐in‐class BTK inhibitor (BTKi) ibrutinib first received US Food and Drug Administration (FDA) approval in 2014 for the treatment of patients with CLL who had received at least one previous therapy, including those with 17p deletion (del(17p)) [1–5]. Venetoclax, a first‐in‐class BCL2 inhibitor (BCL2i), was approved in 2016 for CLL with del(17p) and in 2018 for patients with previously treated CLL [6]. Whereas venetoclax was initially approved as a continuous monotherapy in 2016, the 2018 label expansion approved the fixed‐duration use of venetoclax plus CD20 monoclonal antibody rituximab in relapsed/refractory (R/R) CLL and, in 2019, venetoclax plus obinutuzumab for frontline CLL [7, 8]. This was followed by approvals for the next‐generation BTKi acalabrutinib (in 2019) and zanubrutinib (in 2023) [9, 10].

Despite the positive results achieved with BTKi, many patients with CLL treated with these agents eventually discontinue treatment due to relapse, refractoriness, resistance, or intolerance (R/R/R/I). BTKi treatment intolerance is typically related to adverse events (AEs) such as atrial fibrillation and bleeding [5], and patients who discontinue BTKi because of disease progression tend to have a poorer prognosis. In two large, single‐center studies of patients with CLL treated with BTKis, patients who progressed on BTKi had a median overall survival (OS) of 17.6 and 29.8 months following CLL progression [11, 12]. Many patients with CLL who discontinue BTKi treatment subsequently receive BCL2i‐based regimens [13–15]. However, patients who are treated with at least one BCL2i‐containing regimen (as either mono‐ or combination therapy) in addition to their BTKi regimen and are R/R/R/I to at least one BCL2i‐containing regimen or discontinued BCL2i and had documentation of disease progression in the medical charts—hereafter referred to as “post‐BTKi and post‐BCL2i patients”—have few treatment options and poor prognosis [16–19]. There have been few published studies reporting real‐world clinical outcomes following BTKi treatment discontinuation, and most patients in those studies were heavily pretreated before receiving BTKi [11, 20, 21]. Therefore, less is known about the outcomes of patients with CLL who receive and fail their index BTKi treatment in an earlier line of therapy.

The present study describes patient characteristics, treatment patterns, and real‐world clinical outcomes in a contemporary cohort of patients with CLL who received earlier treatment with BTKi (median of 1 line of therapy before the index BTKi) and were found to be R/R/R/I to the BTKi, including post‐BTKi and post‐BCL2i patients. The correlation between progression‐free survival (PFS) and OS was also assessed in this patient population to validate the utility of PFS as a surrogate for OS.

## 2. Methods

### 2.1. Study Design and Patient Selection

A retrospective, longitudinal chart review was used to examine treatment patterns and clinical outcomes in adult patients with CLL who were determined to be R/R/R/I to BTKi treatment. The study design is illustrated in Supporting Figure S1. The index date was defined as the date of BTKi treatment initiation, as reported in the medical chart. The preindex period was the time from the date of CLL diagnosis to the index date, and the postindex period was the time from the index date to the end of observation, defined as the earliest of death, loss to follow‐up, and end of data availability.

Patients were only included if their medical record documented a confirmed CLL diagnosis, treatment with a BTKi (ibrutinib, acalabrutinib, or other BTKi), and R/R/R/I to BTKi treatment. Refractory and relapsed status was defined per International Workshop on Chronic Lymphocytic Leukemia (iwCLL) guidelines [22], and patients’ status was based on clinicians’ assessment as reported in the patient’s medical chart. Patients were also required to be ≥ 18 years old at the time of CLL diagnosis with ≥ 3 months of clinical documentation before the initiation of the index BTKi treatment and ≥ 12 months of clinical documentation following the initiation of the index BTKi treatment (unless the patient had died). Patients were excluded from the study if they received nonhormonal antineoplastic therapies for other primary malignancies during the pre‐index period.

Two subgroups of patients who were treated with BCL2i, in addition to BTKis, were also analyzed. The double‐exposed subgroup consisted of patients from the overall sample who had been treated with at least one BCL2i‐containing regimen (as either mono‐ or combination therapy) in addition to their BTKi regimen. Additionally, the post‐BTKi and post‐BCL2i subgroup included double‐exposed patients who were R/R/R/I to at least one BCL2i‐containing regimen or who had discontinued BCL2i and had documentation of disease progression in the medical charts.

### 2.2. Data Source

This study assessed clinical outcomes in patients treated at the Dana‐Farber Cancer Institute (DFCI) Center for CLL. A standardized electronic form was used for data abstraction from electronic medical records (EMRs). Data abstraction was conducted between November 2021 and November 2022 for patients who initiated index BTKi treatment at DFCI between July 2011 and September 2019. Data included demographics, oncologic and medical histories, clinical variables (histologic information, secondary malignancies, hematologic characteristics, and laboratory indices), treatments, AEs, reasons for treatment change or discontinuation, and clinical outcomes. Reported diagnostic information included the date of CLL diagnosis, date of R/R/R/I diagnosis, date of entry into care at DFCI, Rai stage, and Binet stage. Genetic data included, but was not limited to, information on chromosomal abnormalities (specifically, del(17p), del(13q), del(11q), and trisomy 12), *IGHV* mutation status, and the presence of somatic mutations (including *PLCγ2*, *BTK*, *IGHV*, *NOTCH1*, *TP53*, *ATM*, *SF3B1*, *BIRC3*, *XPO1*, *BCL6*, and *MYC*) assessed by next‐generation sequencing. Patient data were deidentified and complied with the US Health Insurance Portability and Accountability Act (HIPAA) of 1996. This study received institutional review board (IRB) approval, and all patients consented to DFCI Protocol 99‐224.

### 2.3. Measures and Outcomes

Patient demographics and clinical characteristics were assessed during the pre‐index period. Treatment patterns were assessed in the pre‐ and postindex periods. BTKi treatment patterns included the BTKi received, time from CLL diagnosis to first BTKi treatment, time from BTKi treatment initiation to discontinuation, reason for BTKi treatment discontinuation, and time from BTKi treatment initiation to the next line of treatment. Treatment patterns during lines of therapy after the index BTKi treatment were also assessed and included the proportion of patients using a given treatment in any line of therapy.

Clinical outcomes were evaluated in the post‐index period and included clinicians’ assessment of the first response to index BTKi treatment (complete response, partial response, stable disease, progressive disease with decision to discontinue therapy, and progressive disease with decision to continue therapy for clinical benefit) and overall response rate (ORR; complete and partial response). Additionally, PFS, OS, and the correlation between PFS and OS were also assessed. If available, clinical outcomes of ORR and PFS were evaluated using the criteria from the 2018 iwCLL guidelines [23]. Otherwise, clinician‐assessed response information was extracted verbatim from the treating physician’s notes.

### 2.4. Statistical Analysis

Patient demographics and clinical characteristics were summarized using descriptive statistics including mean (standard deviation [SD]) and median (interquartile range [IQR]) for continuous variables and frequency and proportion for categorical variables. Frequency and proportion were also used to summarize mutation status, treatment sequences and combination therapies, and reasons for treatment discontinuation. Mutation status was reported separately by timing of the mutation (during index BTKi treatment and after discontinuation). A Sankey plot was generated to illustrate the patterns of sequential treatments. Time to discontinuation of the first BTKi and first BCL2i treatment was described with median [IQR]. For clinical outcomes, the proportion of patients by type of treatment response were reported along with ORR, defined as the proportion of patients achieving complete or partial response. PFS and OS were assessed by Kaplan–Meier analysis. For PFS and OS, the time origin was defined as the time of BTKi treatment initiation. Fleischer’s Pearson correlation method was used to assess the correlation between PFS and OS [24]. Clinical outcomes were also assessed for double‐exposed patients and post‐BTKi and post‐BCL2i patients, with the time of BCL2i treatment initiation as the time origin.

## 3. Results

### 3.1. Patient Demographics and Clinical Characteristics

Approximately 300 patients with CLL treated with BTKi at DFCI were initially identified. Of these, 104 were found to be eligible based on the documented confirmation of being R/R/R/I to BTKi and were included in the study population.

Over half of the study population (51.9% [*n* = 54]) received a BTKi in a clinical trial and the remainder as standard of care therapy. The median [IQR] age at index BTKi treatment initiation was 66.7 [60.3–73.0] years, and median [IQR] time from CLL diagnosis to BTKi treatment initiation was 6.7 [2.9–9.4] years (Supporting Table S1). Following BTKi treatment initiation, median [IQR] time to last clinical visit or death was 5.7 [3.3–7.0] years. Thirty‐five patients (33.7%) died (Supporting Table S2).

Of the 101 patients who underwent assessment for cytogenetic abnormalities, 57.4% had del(13q), 27.7% had del(11q), 25.7% had del(17p), and 19.8% had trisomy 12 (Supporting Table S1).

*BTK* mutations were assessed in 31 patients, either during index BTKi treatment or after index BTKi treatment discontinuation. Of the 7 patients assessed during treatment and 24 patients assessed after treatment discontinuation, 4 (57.1%) and 13 (54.2%) had wild type, respectively, and 3 (42.9%) and 11 (45.8%) had mutated *BTK*, respectively. The most frequent mutation was *C481S* (100% of patients with mutations during treatment and 72.2% of patients with mutations after treatment discontinuation), followed by *T474I* (33.3% of patients with mutations during treatment and 27.3% of patients with mutations after treatment discontinuation) and *C481R* (33.3% of patients with mutations during treatment and 18.2% of patients with mutations after treatment discontinuation).

*PLCγ2* mutations were also assessed during index BTKi treatment or after index BTKi treatment discontinuation. *PLCγ2* mutations were present in 1 (14.3%) of the 7 patients screened for this mutation during index BTKi treatment and 2 (9.5%) of the 21 patients screened after treatment discontinuation. Of the 93 patients assessed for other mutations, 49 were assessed before index BTKi initiation, 13 were assessed during index BTKi treatment, and 44 were assessed after discontinuation of index BTKi treatment. The most common other mutations were *TP53, SF3B1, NOTCH1,* and *ATM*. Their prevalence was numerically lowest among patients assessed before BTKi initiation (*TP53*: 30.6%*, SF3B1*: 10.2%*, NOTCH1*: 16.3%, and *ATM*: 8.2%) and trended higher among patients assessed after BTKi initiation (during BTKi treatment [*TP53*: 53.8%, *SF3B1*: 38.5%, *NOTCH1*: 15.4%, and *ATM*: 23.1%]; or after BTKi discontinuation [*TP53*: 43.2%, *SF3B1*: 22.7%, *NOTCH1*: 18.2%, and *ATM*: 15.9%]).

Sixty‐one of the 104 eligible patients (58.7%) were classified as double‐exposed (i.e., were treated with both BTKi and BCL2i). Of these patients, 25 (40.9%) were further subclassified as post‐BTKi and post‐BCL2i (i.e., R/R/R/I to both BTKi and BCL2i). The median [IQR] age at BCL2i treatment initiation was 68.4 [63.3–74.4] years for double‐exposed patients. The median [IQR] time from CLL diagnosis to BCL2i treatment initiation was 9.0 [6.1–12.9] years. Following BCL2i treatment initiation, the median [IQR] time to the last clinical visit or death was 2.6 [1.1–4.4] years (Supporting Table S3). Twenty‐two (36.1%) of the double‐exposed patients died (Supporting Table S4).

### 3.2. Treatment Patterns

**Figure 1:**
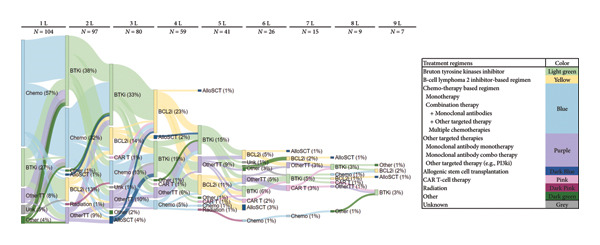
Sankey plot for the overall study population (*N* = 104). Allogeneic hematopoietic cell transplant: AlloSCT; B‐cell lymphoma 2 inhibitor‐based regimen: BCL2i; Bruton’s tyrosine kinase inhibitor: BTKi; chimeric antigen receptor T‐cell therapy: CAR T; chemotherapy: Chemo; other targeted therapy: OtherTT; unknown: Unk. Notes: percentages shown in parentheses represent the proportions out of the overall study population (*N* = 104). For example, 38% of the total 104 patients received a BTKi in the second line of therapy. Chemotherapy‐based regimens include bendamustine; bendamustine + lenalidomide; chlorambucil; chlorambucil + obinutuzumab; chlorambucil + prednisolone; cyclophosphamide + prednisolone + vincristine; fludarabine; fludarabine + alemtuzumab; methylprednisolone + ofatumumab + alemtuzumab; rituximab + bendamustine; rituximab + bendamustine + lenalidomide; rituximab + chlorambucil; rituximab + cyclophosphamide + doxorubicin + vincristine + prednisolone; rituximab + cyclophosphamide + prednisone + vincristine; rituximab + fludarabine; rituximab + fludarabine + chlorambucil; rituximab + fludarabine + cyclophosphamide; rituximab + fludarabine + lenalidomide; rituximab + fludarabine + mitoxantrone; rituximab + fludarabine + obatoclax; rituximab + methylprednisolone; rituximab + pentoxifylline. Other targeted therapies include alemtuzumab; anti‐CD20 bispecific antibody; CD19 trial drug; CD20/CD3ab trial drug; copanlisib + nivolumab; duvelisib; idelalisib; idelalisib + ricolinostat; ipilimumab; nivolumab; obinutuzumab; obinutuzumab + avadomide; obinutuzumab + duvelisib; ofatumumab; ofatumumab + alemtuzumab; ofatumumab + idelalisib; PI3K trial drug; rituximab; rituximab + idelalisib; rituximab + lenalidomide; rituximab + prednisolone; umbralisib; voruciclib.

**Figure 2:**
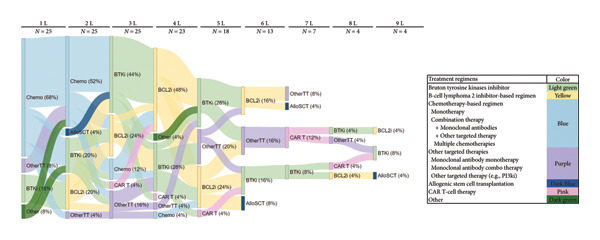
Sankey plot for post‐BTKi and post‐BCL2i patients (*N* = 25). Allogeneic hematopoietic cell transplant: AlloSCT; B‐cell lymphoma 2 inhibitor‐based regimen: BCL2i; Bruton’s tyrosine kinase inhibitor: BTKi; chimeric antigen receptor T‐cell therapy: CAR T; chemotherapy: Chemo; unknown: Unk; other targeted therapy: OtherTT. Notes: percentages shown in parentheses represent the proportions out of cohort of post‐BTKi and post‐BCL2i patients (*N* = 25). For example, 28% of the 25 post‐BTKi and post‐BCL2i patients received a BTKi in the fifth line of therapy. Chemotherapy‐based regimens include chlorambucil; chlorambucil + obinutuzumab; methylprednisolone; rituximab + cyclophosphamide + prednisone + vincristine; rituximab + fludarabine; rituximab + fludarabine + cyclophosphamide; rituximab + fludarabine + mitoxantrone; rituximab + fludarabine + obatoclax. Other targeted therapies include anti‐CD20 bispecific antibody; CD19 trial drug; CD20/CD3ab trial drug; duvelisib; idelalisib; obinutuzumab + avadomide; PI3K trial drug; rituximab; rituximab + lenalidomide; umbralisib.

Most patients (59.9%) received their index BTKi in the first (27.5%) or second (32.4%) line of therapy. Ibrutinib was the most common BTKis (92.3%) and was most often used as monotherapy (59.4%); the remaining 7.7% of patients treated with a BTKi received acalabrutinib as monotherapy (Supporting Table S5). Nearly all patients (98.1%) discontinued their index BTKi, and median [IQR] time to treatment discontinuation was 1.8 [0.6–3.2] years. AEs were the main reason for discontinuation (52.9%), with atrial fibrillation (25.9%) and bleeding events (13.0%) being the most common AEs. Other reasons for discontinuation were disease progression (40.2%) and histologic transformation (2.9%) (Supporting Table S5). After BTKi treatment discontinuation, 59.6% of patients received additional therapies, most commonly venetoclax‐containing regimens (80.6%) as well as other targeted therapies (11%), targeted therapy + monoclonal antibodies (5%), chemotherapy + monoclonal antibodies (7%), and monoclonal antibody monotherapy (7%) (Supporting Table S6 and Figure 1 [Sankey plot]). Most patients (59.0%) received the index BCL2i in the fourth (37.7%) and fifth or later (21.3%) line of therapy. Among double‐exposed patients (who received both BTKi and BCL2i treatments), 73.8% discontinued their first BCL2i treatment after a median [IQR] time of 0.8 [0.2–2.1] years (Supporting Table S7). Figure 2 reports the treatment lines of post‐BTKi post‐BCL2i patients (i.e., patients who were R/R/R/I to both BTKis and BCL2is).

### 3.3. Clinical Outcomes

Of the 84 patients with documented clinical assessment of first response to their index BTKi, 16.7% achieved a clinical complete response and 66.7% a partial response; 4.8% had stable disease, 4.8% had progressive disease with decision to discontinue therapy, and 2.4% had progressive disease with decision to continue therapy for clinical benefit. The ORR to the index BTKi among all patients with documented clinical assessment of first response was therefore 83.3% (Table 1). Of the 45 double‐exposed patients with known clinician’s assessment of first response to BCL2i, following treatment with BTKi, 35.6% achieved a clinical complete response and 42.2% had a partial response; 8.9% had stable disease and 6.7% had progressive disease with decision to discontinue therapy (Table 2). The ORR to second treatment at the first response assessment was therefore 77.8% among double‐exposed patients. Of the 20 post‐BTKi and post‐BCL2i patients (i.e., patients with documentation of being R/R/R/I to both BTKi and BCL2i) with a known clinician’s assessment of the first response to BCL2i, following treatment with BTKis, 30.0% achieved a clinical complete response, 55.0% achieved a partial response, and 15.0% had progressive disease with decision to discontinue therapy (Table 2). Among post‐BTKi post‐BCL2i patients who received BCL2i as their second treatment, ORR to BCL2i at first assessment was therefore 85.0%.

**Table 1:** Summary of response to index BTKi treatment^1^.

	Overall
	*N* = 104
Patients with an assessment of first response to index BTKi, *n* ^1,2^	84 (80.8)
Time from index date to date of clinician’s assessment of first response to index BTKi, months^1,3^	
Median [Q1, Q3]	6.7 [2.8, 15.0]
Clinical parameters considered in informing assessment of first response to index BTKi, *n* (%)^1^	
Known	81 (96.4)
Circulating lymphocyte count	71 (87.7)
Constitutional symptoms	65 (80.2)
Hemoglobin level	69 (85.2)
Marrow analysis	10 (12.3)
Platelet count	71 (87.7)
Size of liver and spleen	55 (67.9)
Size of lymph nodes	71 (87.7)
Other	4 (4.9)
Unknown	3 (3.6)
Clinician’s assessment of first response to index BTKi, *n* (%)^1^	
Known	84 (100.0)
Clinician‐assessed complete response	14 (16.7)
Clinician‐assessed partial response	56 (66.7)
Clinician‐assessed stable disease	4 (4.8)
Progressive disease, with decision to discontinue therapy	4 (4.8)
Progressive disease, with decision to continue therapy for clinical benefit	2 (2.4)
Other	4 (4.8)
Unknown	0 (0.0)
Overall response rate to index BTKi, *n* (%)^1,4^	70 (83.3)

*Note: N*: sample size; Q1: first quartile; Q3: third quartile.

Abbreviations: BTKi = Bruton’s tyrosine kinase inhibitor, SD = standard deviation.

^1^The index BTKi was the first BTKi the patient was treated with.

^2^Patients were excluded from the analysis if the date of the clinician’s assessment of first response to index BTKi was missing or if treatment response was assessed after treatment discontinuation.

^3^The index date was defined as the initiation of the first treatment with a BTKi.

^4^The overall response rate was proportion of patients with clinician‐assessed complete response or clinician‐assessed partial response.

**Table 2:** Summary of response to BTKi/BCL2i treatment in double‐exposed and post‐BTKi and post‐BCL2i patients^1^.

	Double‐exposed	Post‐BTKi and post‐BCL2i
	*N* = 61	*N* = 25
Patients with an assessment of the first response to subsequent BTKi or BCL2i, *n* ^1,2^	52 (85.2)	25 (100.0)
Patients with BTKi before first BCL2i	48 (92.3)	21 (84.0)
Patients with BCL2i before first BTKi	4 (7.7)	4 (16.0)
Response to first BCL2i treatment following BTKi	48 (92.3)	21 (84.0)
Time from the treatment initiation to the date of clinician’s assessment of the first response to BCL2is, months		
Median [Q1, Q3]	5.9 [2.9, 15.6]	5.9 [2.7, 9.7]
Clinical parameters considered in informing the assessment of the first response to BCL2i, *n* (%)		
Known	45 (93.8)	20 (95.2)
Circulating lymphocyte count	40 (88.9)	17 (85.0)
Constitutional symptoms	36 (80.0)	16 (80.0)
Hemoglobin level	37 (82.2)	16 (80.0)
Marrow analysis	8 (17.8)	2 (10.0)
Platelet count	35 (77.8)	15 (75.0)
Size of liver and spleen	30 (66.7)	14 (70.0)
Size of lymph nodes	39 (86.7)	19 (95.0)
Other	6 (13.3)	2 (10.0)
Unknown	3 (6.3)	1 (4.8)
Clinician’s assessment of the first response to BCL2i, *n* (%)		
Known	45 (93.8)	20 (95.2)
Clinician‐assessed complete response	16 (35.6)	6 (30.0)
Clinician‐assessed partial response	19 (42.2)	11 (55.0)
Clinician‐assessed stable disease	4 (8.9)	0 (0.0)
Progressive disease, decision to discontinue therapy	3 (6.7)	3 (15.0)
Other	3 (6.7)	0 (0.0)
Unknown	3 (6.3)	1 (4.8)
Response to first BTKis treatment following BCL2i	4 (100.0)	4 (100.0)
Time from the treatment initiation to the date of clinician’s assessment of the first response to BTKis, months		
Median [Q1, Q3]	7.5 [3.9, 11.1]	7.5 [3.9, 11.1]
Clinical parameters considered in informing the assessment of the first response to BTKi, *n* (%)		
Known	3 (75.0)	3 (75.0)
Circulating lymphocyte count	2 (66.7)	2 (66.7)
Constitutional symptoms	3 (100.0)	3 (100.0)
Hemoglobin level	3 (100.0)	3 (100.0)
Marrow analysis	0 (0.0)	0 (0.0)
Platelet count	3 (100.0)	3 (100.0)
Size of liver and spleen	2 (66.7)	2 (66.7)
Size of lymph nodes	3 (100.0)	3 (100.0)
Other	0 (0.0)	0 (0.0)
Unknown	1 (25.0)	1 (25.0)
Clinician’s assessment of the first response to BTKi, *n* (%)		
Known	4 (100.0)	4 (100.0)
Clinician‐assessed complete response	1 (25.0)	1 (25.0)
Clinician‐assessed partial response	3 (75.0)	3 (75.0)
Overall response rate to BTKis, *n* (%)^3^	4 (100.0)	4 (100.0)

*Note: N*: sample size; Q1: first quartile; Q3: third quartile.

Abbreviations: BCL2i = B‐cell lymphoma 2 inhibitor, BTKi = Bruton’s tyrosine kinase inhibitor, SD = standard deviation.

^1^This analysis reports the first response to the second treatment of either BTKis or BCL2is in double‐exposed patients. If the patient had two consecutive lines of therapy with BTKi followed by another line of therapy with BCL2is, this analysis will report responses for the BCL2i line of therapy. The results are presented in two parts: firstly showing patients who received BTKis prior to BCL2i (*N* = 48 [92.3%]) and then results for patients who received BCL2is prior to BTKi (*N* = 4 [7.7%]).

^2^Patients were excluded from the analysis if the date of the clinician’s assessment of first response to treatment was missing.

^3^Overall response rate was the proportion of patients with clinician‐assessed complete response or clinician‐assessed partial response.

^4^Data were analyzed for the patients with an assessment of the first response to treatment.

Among the overall study population (patients with CLL who were R/R/R/I to BTKi), the median (95% CI) PFS was 3.2 (2.6, 3.9) years, and the median (95% CI) OS was 8.9 (6.7, NR) years from index BTKi initiation. The correlation (95% CI) between PFS and OS was 0.79 (0.63, 0.94) (Supporting Table S8).

## 4. Discussion

Covalent BTKi have transformed the management of CLL by significantly prolonging survival [25], but many patients who receive BTKi eventually discontinue the treatment due to intolerance or disease progression. Most of these patients receive additional lines of therapy, which are often BCL2i‐containing regimens. However, a subset of patients treated with BTKis and subsequently with BCL2i eventually progress and require another line of therapy, representing a patient population with unmet clinical need. The current study from a large US cancer center evaluated the characteristics, treatment patterns, and clinical outcomes of a cohort of these patients who were treated with BTKi and subsequently discontinued the treatment.

Although BTKi generally have a favorable safety profile, AEs are the most common reason for BTKi treatment discontinuation [11, 19–21, 26–29]. In a previous real‐world study, approximately 30% of patients with CLL treated with ibrutinib had discontinued the treatment within 2 years, mostly because of toxicity (56%); a smaller proportion discontinued because of disease progression (32.0%) [21]. Other studies of ibrutinib have also reported AEs or treatment toxicity as the most common reason for treatment discontinuation, followed by disease progression [11, 20, 30]. AEs were also the main reason for BTKi treatment discontinuation in our study (52.9% of patients) including atrial fibrillation as the most common one (25.9% of the patients discontinuing because of AEs), followed by disease progression (40.2%). Notably, this pattern was observed even though the majority of patients received their index BTKi treatment in the first or second line of therapy, in contrast to the aforementioned studies in which patients had received a median of two to three lines of therapy before their index BTKi [11, 20, 21, 30]. Thus, toxicities associated with BTKi can lead to treatment discontinuation in any line of therapy, thereby preventing some patients from achieving disease control even in earlier lines of therapy.

The poor prognosis of patients in our study following the discontinuation of the index BTKi is suggested by the observed changes in the mutation profile. Among patients with assessment of mutation status after discontinuation, C481S was the most frequently detected *BTK* mutation (72%). *BTK* C481S and *PLCγ2* mutations are known to be present at high frequencies in patients who have relapsed after treatment with ibrutinib [31, 32] and acalabrutinib [33] and may confer resistance to these therapies. Our analysis of mutations focused on the periods during BTKi treatment and after BTKi discontinuation. Further research is warranted to evaluate mutation status relative to treatment lines following BTKi discontinuation.

Following BTKi treatment discontinuation, the majority of patients in our study (59.6%) received a subsequent line of therapy, most often a venetoclax‐containing regimen (80.6%). This is in line with the reported tolerability and clinical activity of venetoclax in patients previously treated with BTKi [12, 14, 34–36]. In a Phase 2 trial of venetoclax in patients with relapsed/refractory (R/R) CLL with prior BTKi treatment, venetoclax was welltolerated and the ORR was 65% over a median follow‐up of 14 months [14]. It was later confirmed that venetoclax monotherapy yielded durable responses (median PFS of 24.7 months) in patients with previous exposure to ibrutinib, irrespective of *BTK* mutation status [37]. In patients with CLL who progressed on ibrutinib, treatment with venetoclax was associated with a longer OS than chemoimmunotherapy (CIT), phosphoinositide 3 kinase inhibitor (PI3Ki), or anti‐CD20 monoclonal antibody treatment [12, 34]. However, patients with CLL who experience disease progression after both BTKi and BCL2i treatment have poor outcomes [16–19]: the median OS in this population has been reported as 3.6 months [18] and 21.2 months [16], and patients who initiated a subsequent line of therapy had a median PFS of 6.8 months [16] and 9.2 months [19]. Moreover, patients with CLL or small lymphocytic lymphoma who discontinued both covalent BTKi and BCL2i treatment had a median time from discontinuation of these therapies to discontinuation of the next treatment or death of 5.6 months [17]. In our study, the post‐BTKi and post‐BCL2i subgroup had a 44.0% rate of mortality following initiation of the second treatment and a 34.0% rate of mortality in the overall study sample (following initiation of the index BTKi). The rate of progressive disease was 15.0% following initiation of the second treatment and 7.2% after the index BTKi. These results are consistent with the progressive worsening prognosis of double‐exposed patients and the need for more effective treatment options for this population. More effective treatment options may also be needed for the 40.4% of patients in our study who received no further treatment after BTKi discontinuation. While the scope of this study was to analyze patients receiving follow‐up treatments (and, specifically, BCL2i), further research may help better understand the unmet treatment needs of patients who do not receive additional therapies after BTKi discontinuation.

Until recently, outside of clinical trials, the only available treatments for patients who progressed after or were intolerant to BTKi and BCL2i were allogeneic hematopoietic cell transplantation (alloHCT), PI3Ki, alemtuzumab, and CIT. Recently, newly approved therapies have become available for this difficult‐to‐treat population. In December 2023, the noncovalent BTKi pirtobrutinib was granted accelerated approval by the FDA for the treatment of adults with CLL who previously received ≥ 2 lines of therapy including a BTKi and BCL2i. The approval was based on the results from the Phase 1/2 BRUIN trial, which showed an ORR of 73.3% with pirtobrutinib in patients with CLL previously exposed to a covalent BTKi (vs. 70.0% in patients who previously received both BTKi and BCL2i) and median PFS of 19.6 months (vs. 16.8 months in the double‐exposed population) [38]. In March 2024, the CD19‐directed CAR T‐cell therapy lisocabtagene maraleucel (liso‐cel) received accelerated approval for the treatment of R/R CLL following ≥ 2 lines of therapy including a BTKi and BCL2i. The approval was based on data from the single‐arm Phase 1/2 TRANSCEND CLL 004 trial, where the ORR was 45%, the CR rate was 18%, and the median duration of response was 35.3 months [39]. Longer follow‐up is needed to ascertain the long‐term clinical benefits with pirtobrutinib and liso‐cel, especially as mutations conferring resistance to pirtobrutinib (e.g., in the kinase domain of *BTK* and in the *BTK* target *PLCγ2*) have already been identified [40, 41]. With the discovery of these mutations, and given the low CR rate observed with liso‐cel, there is considerable interest in other promising novel therapies such as the non‐covalent BTKi nemtabrutinib, BTK degraders (e.g., NX‐5948 and BGB‐16673), and bispecific T‐cell engagers that are currently under investigation [42].

In addition to examining treatment patterns and clinical outcomes of patients with CLL who were R/R/R/I to BTKi, another objective of this study was to investigate whether PFS can be used as a surrogate endpoint for OS when evaluating treatment effectiveness. In a meta‐analysis of 23 studies in CLL (mostly of patients with refractory or progressive disease), median PFS and median OS were highly significantly correlated (*r* = 0.813) [43]. We found a similar and significant positive correlation (*r* = 0.79) between PFS and OS measured from initiation of first BTKi, in patients with CLL who were R/R/R/I to BTKi, supporting the surrogacy of PFS for OS in this population. This finding provides further evidence that PFS remains the preferred endpoint for the regulatory approval of new drugs in CLL given the long‐time horizon to capture OS data.

The results of this study should be viewed in the context of certain limitations. First, because of the retrospective and observational nature of the study, patient selection, disease characteristics, treatments, and study endpoints were limited by data availability; detailed information may not have been available for some variables and the level of detail varied across patients. Second, only the clinical assessment of first response to the index BTKi was analyzed; requiring a confirmed response by including the subsequent clinical assessments would likely have resulted in lower response rates. Finally, as all patients included in the analysis were treated at a large cancer center, the findings may not be generalizable to all patients with CLL treated in community‐based practice settings.

## 5. Conclusions

In a contemporary cohort of patients with CLL who were R/R/R/I to BTKi in the United States, BTKi treatment discontinuation was mainly due to AEs or disease progression. One‐quarter of patients switched to a second BTKi and more than half received subsequent therapies, mostly BCL2i‐containing regimens. Over 70% of patients receiving BCL2i after BTKi had a complete or partial response. However, > 70% of double‐exposed patients eventually discontinued BCL2i treatment and more than one‐third died, with a median follow‐up of 2.6 years after BCL2i initiation. These findings underscore the clinical challenge of managing the growing population of patients with CLL who have been treated with BTKi and subsequently received BCL2i‐containing regimens. Even with the recent approvals of pirtobrutinib and liso‐cel, the evolving therapeutic need of this emerging patient population necessitates continued investigation and improved treatment options. Novel therapies now in development are offering hope for better options on the horizon that may reshape the R/R CLL treatment landscape.

## Ethics Statement

Data were deidentified and comply with HIPAA patient requirements; therefore, no review by an IRB was required per Title 45 of CFR, Part 46.101(b) (4) (https://www.hhs.gov/ohrp/regulations-and-policy/regulations/45-cfr-46/#46.101).

## Disclosure

Part of the material in this article was previously presented, including as a poster at the 64th American Society of Hematology Annual Meeting and Exposition (December 10–13, 2022; New Orleans, LA); as a poster at the 2023 American Society of Clinical Oncology Annual Meeting (June 2, 2023; online); and as two posters at the 65th American Society of Hematology Annual Meeting and Exposition (December 9–12, 2023; San Diego, CA). All authors approved the version of the article to be published and agree to be accountable for all aspects of the work. The study sponsor was involved in several aspects of the research including study design, data interpretation, manuscript writing, and decision to submit the manuscript for publication.

## Conflicts of Interest

Xiaoqin Yang, Mohammed Z. H. Farooqui, Enrico De Nigris, and Shravanthi R. Gandra are employees of Merck & Co., Inc. Xiaoqin Yang and Enrico De Nigris own Merck & Co., Inc. stock/stock options.

Lynn Huynh, Enrico Zanardo, and Mei Sheng Duh are employees of Analysis Group, Inc., a consulting company that has provided paid consulting services to Merck & Co., Inc., which funded the development and conduct of this study and manuscript.

Kevin H. Lin has received support for conference travel from Brigham and Women’s Hospital.

Jennifer R. Brown has received research grants from BeiGene, Gilead, iOnctura, Loxo/Lilly, MEI Pharma, and TG Therapeutics; royalties from UpToDate; and consultancy fees from AbbVie, Acerta/AstraZeneca, Alloplex Biotherapeutics, BeiGene, Bristol‐Myers Squibb, EcoR1, Galapagos NV, Genentech/Roche, Grifols Worldwide Operations, InnoCare Pharama Inc., iOnctura, Kite Pharma, Loxo/Lilly, Magnet Biomedicine, Merck, Numab Therapeutics, Pfizer, and Pharmacylics; and participated on a board for Grifols Therapeutics.

Matthew S. Davids has received research grants from Ascentage Pharma, MEI Pharma, and Novartis; royalties from UpToDate; and consultancy fees from AbbVie, Adaptive Biotechnologies, Ascentage Pharma, AstraZeneca, BeiGene, Bristol‐Myers Squibb, Eli Lilly, Galapagos, Genentech, Genmab, Janssen, Merck, MEI Pharma, Nuvalent, SecuraBio, Schrödinger Therapeutics Group, Takeda, and TG Therapeutics; and participated on the CLL‐17 Safety Monitoring Board.

The other authors declare no conflicts of interest.

## Author Contributions

All authors were involved in study conception and design or data analysis and interpretation, and drafting or critically revising the article for intellectual content.

## Funding

This study was funded by Merck & Co., Inc.

## Supporting Information

Additional supporting information can be found online in the Supporting Information section.

## Supporting information


**Supporting Information 1** Supporting Figure S1: Study design scheme.

**Supporting Information 2** Supporting Table S1: Demographic and clinical characteristics.

**Supporting Information 3** Supporting Table S2: Summary of mortality.

**Supporting Information 4** Supporting Table S3: Demographic and clinical characteristics of double‐exposed and post‐BTKi and post‐BCL2i patients.

**Supporting Information 5** Supporting Table S4: Summary of mortality for double‐exposed and post‐BTKi and post‐BCL2i patients.

**Supporting Information 6** Supporting Table S5: Summary of BTKi treatment and reasons for discontinuation of index BTKi.

**Supporting Information 7** Supporting Table S6: Treatments following last BTKi treatment.

**Supporting Information 8** Supporting Table S7: Summary of BTKi and BCL2i lines of therapy in double‐exposed and post‐BTKi and post‐BCL2i patients.

**Supporting Information 9** Supporting Table S8: Progression‐free survival and overall survival correlation analysis.

## Data Availability

Data are not available due to legal restrictions. Due to the nature of this research, participants of this study did not agree for their data to be shared publicly, so supporting data are not available. Therefore, restrictions apply to the availability of these data, which are not publicly available.
