# Supplementary material for: Clinical Outcomes in Patients With CLL Treated With BTKi at a Large US Cancer Center
Source: Adv Hematol. 2025 Nov 30;2025:7492594. doi: 10.1155/ah/7492594 (PMC12665162; doi:10.1155/ah/7492594)
Supplement: Supplementary file 1 — Supporting Information 1 Supporting Figure S1: Study design scheme. [file AH-2025-7492594-s008.pdf]

Supplemental Figure S1. Study design scheme

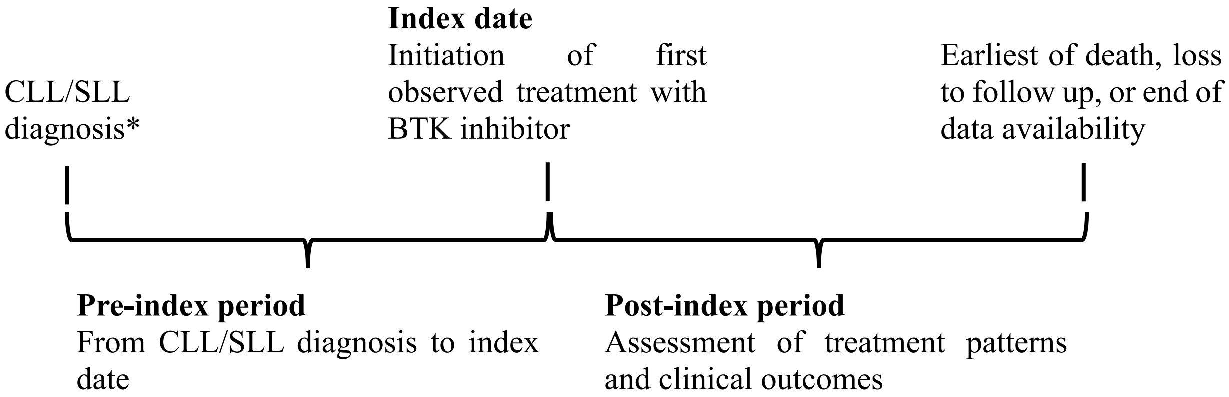

*CLL/SLL diagnosis may precede the date of entry into care at DFCI
