# Supplementary material for: Clinical Outcomes in Patients With CLL Treated With BTKi at a Large US Cancer Center
Source: Adv Hematol. 2025 Nov 30;2025:7492594. doi: 10.1155/ah/7492594 (PMC12665162; doi:10.1155/ah/7492594)
Supplement: Supplementary file 2 — Supporting Information 2 Supporting Table S1: Demographic and clinical characteristics. [file AH-2025-7492594-s003.pdf]

Supplemental Table S1. Demographic and clinical characteristics

	**Overall N = 104**
**Age at index BTKi treatment initiation, years[1]**	
Median [Q1, Q3]	66.7 [60.3, 73.0]
**Age at first CLL diagnosis, years**	
Median [Q1, Q3]	59.7 [53.0, 65.1]
**Time from CLL diagnosis to index BTKi treatment initiation, years[1]**	
Median [Q1, Q3]	6.7 [2.9, 9.4]
**Time from index BTKi treatment initiation to last clinical visit or death, years[1]**	
Median [Q1, Q3]	5.7 [3.3, 7.0]
**Sex, n (%)**	
Known	104 (100.0)
Male	66 (63.5)
Female	38 (36.5)
Other	0 (0.0)
Unknown	0 (0.0)
**Race, n (%)**	
Known	101 (97.1)
White	101 (100.0)
Black or African American	1 (1.0)
Asian	0 (0.0)
Native American or American Indian	0 (0.0)
Native Hawaiian or Other Pacific Islander	0 (0.0)
Other	0 (0.0)
Unknown	3 (2.9)
**Ethnicity, n (%)**	
Known	98 (94.2)
Hispanic	3 (3.1)
Non-Hispanic	95 (96.9)
Unknown	6 (5.8)
**Rai stage at index date, n (%)[1]**	
Assessed	82 (78.8)
Rai stage by category	
Low (stage 0-2)	36 (43.9)
High (stage 3-4)	46 (56.1)
Not assessed/unknown[2]	22 (21.2)
**Type of cytogenetic abnormalities, n (%)**	
Assessed[3]	101 (97.1)
Trisomy 12	20 (19.8)
Del(11q) / 11q-	28 (27.7)
Del(13q) / 13q-	58 (57.4)
Del(17p) / 17p-	26 (25.7)
Del(6q) / 6q-	3 (3.0)
Other	11 (10.9)

Not assessed/unknown	3 (2.9)
**TP53 mutation, n (%)**	
Known	58 (55.8)
Positive	38 (65.5)
Negative	20 (34.5)
Not assessed/unknown	46 (44.2)
**Patients with del(17p) assessment, n (%)[4]**	101 (97.1)
Del(17p) and TP53	17 (16.8)
Del(17p) or TP53	40 (39.6)
**IGHV mutation, n (%)**	
Assessed	83 (79.8)
≥ 2% mutated	21 (25.3)
< 2% mutated	62 (74.7)
Other IGHV mutation status	1 (1.2)
Not assessed/unknown	21 (20.2)

Abbreviations: BTKi: Bruton's tyrosine kinase inhibitor; CLL: chronic lymphocytic leukemia; IGHV: immunoglobulin heavy chain gene; mg/L: milligrams per liter; N: sample size; SD: standard deviation; SLL: small lymphocytic lymphoma; U/L: units per liter; Q1: first quartile; Q3: third quartile.

Notes:
[1] The index BTKi is the first BTKi the patient was treated with.
[2] The patient either did not have a Rai stage assessment or had a Rai stage assessed but the results are not provided in the DFCI medical chart as it could have been performed at another center.
[3] Multiple abnormalities could be selected.
[4] 'Del(17p) and TP53' and 'Del(17p) or TP53' are analyzed among the 101 patients with an assessment for del(17p).
