# Supplementary material for: Clinical Outcomes in Patients With CLL Treated With BTKi at a Large US Cancer Center
Source: Adv Hematol. 2025 Nov 30;2025:7492594. doi: 10.1155/ah/7492594 (PMC12665162; doi:10.1155/ah/7492594)
Supplement: Supplementary file 3 — Supporting Information 3 Supporting Table S2: Summary of mortality. [file AH-2025-7492594-s001.pdf]

Supplemental Table S2. Summary of mortality

	**Overall N = 104**
**Mortality, n (%)**	
Alive	69 (66.3)
Dead	35 (33.7)
**Age at death, years[1]**	
Median [Q1, Q3]	70.9 [65.0, 79.0]
**Primary cause of death, n (%)[1]**	
Known	29 (82.9)
Disease progression	18 (62.1)
Treatment-related toxicity	0 (0.0)
Infection	5 (17.2)
Secondary malignancy	2 (6.9)
Other	4 (13.8)
Unknown	6 (17.1)

Abbreviations: N: sample size; Q1: first quartile; Q3: third quartile; SD: standard deviation.

Notes:
[1] Data was analyzed for the 35 patients who died.
