# Supplementary material for: Clinical Outcomes in Patients With CLL Treated With BTKi at a Large US Cancer Center
Source: Adv Hematol. 2025 Nov 30;2025:7492594. doi: 10.1155/ah/7492594 (PMC12665162; doi:10.1155/ah/7492594)
Supplement: Supplementary file 4 — Supporting Information 4 Supporting Table S3: Demographic and clinical characteristics of double‐exposed and post‐BTKi and post‐BCL2i patients. [file AH-2025-7492594-s009.pdf]

**Supplemental Table S3. Demographic and clinical characteristics of double-exposed and post-BTKi and post-BCL2i patients**

| | Double-exposed<br>N = 61 | Post-BTKi and post-BCL2i<br>N = 25 |
|---|---|---|
| **Age at first BTKi treatment initiation, years** | | |
| Mean ± SD | 65.0 ± 9.3 | 64.2 ± 8.5 |
| Median [Q1, Q3] | 66.3 [60.1, 71.3] | 65.2 [59.4, 70.2] |
| **Age at first BCL2i treatment initiation, years** | | |
| Mean ± SD | 67.8 ± 9.4 | 66.3 ± 8.3 |
| Median [Q1, Q3] | 68.4 [63.3, 74.4] | 65.6 [63.2, 72.2] |
| **Age at first CLL diagnosis, years** | | |
| Median [Q1, Q3] | 60.5 [52.7, 64.6] | 60.8 [52.1, 64.6] |
| **Time from CLL diagnosis to first BTKi treatment initiation, years** | | |
| Median [Q1, Q3] | 6.0 [3.0, 8.3] | 6.0 [2.9, 8.6] |
| **Time from CLL diagnosis to first BCL2i treatment initiation, years** | | |
| Median [Q1, Q3] | 9.0 [6.1, 12.9] | 8.5 [4.8, 11.6] |
| **Time from first BTKi treatment initiation to last clinical visit or death, years** | | |
| Median [Q1, Q3] | 6.0 [3.9, 7.6] | 6.0 [4.2, 7.7] |
| **Time from first BCL2i treatment initiation to last clinical visit or death, years** | | |
| Median [Q1, Q3] | 2.6 [1.1, 4.4] | 3.4 [2.3, 5.4] |
| **Sex, n (%)** | | |
| Known | 61 (100.0) | 25 (100.0) |
| Male | 42 (68.9) | 16 (64.0) |
| Female | 19 (31.1) | 9 (36.0) |
| **Race, n (%)** | | |
| Known | 59 (96.7) | 24 (96.0) |
| White | 59 (100.0) | 24 (100.0) |
| Black or African American | 0 (0.0) | 0 (0.0) |
| Asian | 0 (0.0) | 0 (0.0) |
| Native American or American Indian | 0 (0.0) | 0 (0.0) |
| Native Hawaiian or Other Pacific Islander | 0 (0.0) | 0 (0.0) |
| Other | 0 (0.0) | 0 (0.0) |
| Unknown | 2 (3.3) | 1 (4.0) |
| **Ethnicity, n (%)** | | |
| Known | 60 (98.4) | 25 (100.0) |
| Hispanic | 3 (5.0) | 2 (8.0) |
| Non-Hispanic | 57 (95.0) | 23 (92.0) |
| Unknown | 1 (1.6) | 0 (0.0) |
| **Rai stage at index date, n (%)** | | |
| Assessed | 46 (75.4) | 19 (76.0) |
| Rai stage by category | | |
| Low (stage 0-2) | 20 (43.5) | 9 (47.4) |
| High (stage 3-4) | 26 (56.5) | 10 (52.6) |

| | | |
|---|---|---|
| Not assessed/unknown[1] | 15 (24.6) | 6 (24.0) |
| **Type of cytogenetic abnormalities, n (%)** | | |
| Assessed[2] | 58 (95.1) | 23 (92.0) |
| Trisomy 12 | 9 (15.5) | 4 (17.4) |
| Del(11q) / 11q- | 15 (25.9) | 5 (21.7) |
| Del(13q) / 13q- | 32 (55.2) | 11 (47.8) |
| Del(17p) / 17p- | 21 (36.2) | 9 (39.1) |
| Del(6q) / 6q- | 2 (3.4) | 0 (0.0) |
| Other | 7 (12.1) | 1 (4.3) |
| Not assessed/unknown | 3 (4.9) | 2 (8.0) |
| **TP53 mutation, n (%)** | | |
| Known | 37 (60.7) | 16 (64.0) |
| Positive | 28 (75.7) | 14 (87.5) |
| Negative | 9 (24.3) | 2 (12.5) |
| Not assessed/unknown | 24 (39.3) | 9 (36.0) |
| **Patients with del(17p) assessment, n (%)[3]** | 58 (95.1) | 23 (92.0) |
| Del(17p) and TP53 | 14 (24.1) | 6 (26.1) |
| Del(17p) or TP53 | 29 (50.0) | 14 (60.9) |
| **IGHV mutation, n (%)** | | |
| Assessed | 49 (80.3) | 20 (80.0) |
| ≥ 2% mutated | 8 (16.3) | 1 (5.0) |
| < 2% mutated | 41 (83.7) | 19 (95.0) |
| Other IGHV mutation status | 1 (2.0) | 0 (0.0) |
| Not assessed/unknown | 12 (19.7) | 5 (20.0) |

**Abbreviations:** BCL2i: B-cell lymphoma 2 inhibitor; BTKi: Bruton's tyrosine kinase inhibitor; CLL: chronic lymphocytic leukemia; IGHV: immunoglobulin heavy chain gene; mg/L: milligrams per liter; N: sample size; SD: standard deviation; SLL: small lymphocytic lymphoma; U/L: units per liter; Q1: first quartile; Q3: third quartile.

**Notes:**

[1] The patient either did not have a Rai stage assessment, or had a Rai stage assessed but the results are not provided in the DFCI medical chart as it could have been performed at another center.

[2] Multiple abnormalities could be selected.

[3] 'Del(17p) and TP53' and 'Del(17p) or TP53' are analyzed among the patients with an assessment for del(17p).
