# Supplementary material for: Clinical Outcomes in Patients With CLL Treated With BTKi at a Large US Cancer Center
Source: Adv Hematol. 2025 Nov 30;2025:7492594. doi: 10.1155/ah/7492594 (PMC12665162; doi:10.1155/ah/7492594)
Supplement: Supplementary file 5 — Supporting Information 5 Supporting Table S4: Summary of mortality for double‐exposed and post‐BTKi and post‐BCL2i patients. [file AH-2025-7492594-s006.pdf]

Supplemental Table S4. Summary of mortality for double-exposed and post-BTKi and post-BCL2i patients

	**Double-exposed N = 61**	**Post-BTKi and post-BCL2i N = 25**
**Mortality, n (%)**		
Alive	39 (63.9)	14 (56.0)
Dead	22 (36.1)	11 (44.0)
Unknown	0 (0.0)	0 (0.0)
**Age at death, years[1]**		
Median [Q1, Q3]	68.6 [65.0, 74.3]	69.3 [65.1, 74.3]
**Primary cause of death, n (%)[1]**		
Known	18 (81.8)	8 (72.7)
Disease progression	11 (61.1)	5 (62.5)
Secondary malignancy	2 (11.1)	2 (25.0)
Infection	2 (11.1)	0 (0.0)
Treatment-related toxicity	0 (0.0)	0 (0.0)
Other	3 (16.7)	1 (12.5)
Unknown	4 (18.2)	3 (27.3)

Abbreviations: N: sample size; Q1: first quartile; Q3: third quartile; SD: standard deviation.

Notes:
[1] Data was analyzed for the patients who died.
