# Supplementary material for: Clinical Outcomes in Patients With CLL Treated With BTKi at a Large US Cancer Center
Source: Adv Hematol. 2025 Nov 30;2025:7492594. doi: 10.1155/ah/7492594 (PMC12665162; doi:10.1155/ah/7492594)
Supplement: Supplementary file 6 — Supporting Information 6 Supporting Table S5: Summary of BTKi treatment and reasons for discontinuation of index BTKi. [file AH-2025-7492594-s002.pdf]

Supplemental Table S5. Summary of BTKi treatment and reasons for discontinuation of index BTKi[1]

	**Overall N = 104**
**Index BTKi regimens, n (%)[1]**	
**Ibrutinib**	96 (92.3)
Ibrutinib monotherapy	57 (59.4)
Ibrutinib+CD20 mAB	11 (11.5)
Ibrutinib+rituximab	2 (2.1)
**Acalabrutinib**	8 (7.7)
Acalabrutinib monotherapy	8 (100.0)
Acalabrutinib+CD20 mAB	0 (0.0)
**Index BTKi line of therapy, n (%)[1]**	
Known	102 (98.1)
1	28 (27.5)
2	33 (32.4)
3	23 (22.5)
4	12 (11.8)
≥ 5	6 (5.9)
Unknown	2 (1.9)
**Index BTKi treatment discontinuation, n (%)[1]**	
Still on index BTKi[3]	2 (1.9)
Discontinued index BTKi	102 (98.1)
**Time from index date to index BTKi treatment discontinuation, years[1,4]**	
Median [Q1, Q3]	1.8 [0.6, 3.2]
**Reason for index BTKi treatment discontinuation, n (%)[1]**	
Discontinued index BTKi	102 (98.1)
Toxicity/adverse events[5,6]	54 (52.9)
Hemorrhage/bleeding	7 (13.0)
Atrial fibrillation	14 (25.9)
Anemia	2 (3.7)
Arthralgia	4 (7.4)
Thrombocytopenia	3 (5.6)
Bruising	2 (3.7)
Diarrhea	4 (7.4)
Fatigue	2 (3.7)
Burning	0 (0.0)
Cough	0 (0.0)
Headache	0 (0.0)
Infection	1 (1.9)
Myalgia	2 (3.7)
Nausea	1 (1.9)
Neutropenia	0 (0.0)
Pyrexia	0 (0.0)
Rash	4 (7.4)

Upper respiratory tract infection	1 (1.9)
Other	24 (44.4)
Secondary malignancy	1 (1.0)
CLL progression	41 (40.2)
Histologic transformation	3 (2.9)
Eligibility for clinical trial treatment	1 (1.0)
Patient preference	0 (0.0)
Financial toxicity	0 (0.0)
Other[7]	2 (2.0)
Unknown	0 (0.0)

Abbreviations: 1L: first-line therapy; 2L: second-line therapy; 3L: third-line therapy; 4L: fourth line of therapy; 5L+: fifth line of therapy and after; BTKi: Bruton's tyrosine kinase inhibitor; CAR: chimeric antigen receptor; CLL: chronic lymphocytic leukemia; mab: monoclonal antibodies; N: sample size; SD: standard deviation; SLL: small lymphocytic lymphoma; Q1: first quartile; Q3: third quartile.

Notes:
[1] The index BTKi is the first BTKi the patient was treated with.
[2] Two patients with unknown index BTKi line of therapy were excluded from the analyses by line of therapy.
[3] Of the two patients who are still on BTKi, one is enrolled in a clinical trial that is ongoing and the other was lost to follow-up.
[4] The index date is defined as the initiation of the first treatment with a BTKi.
[5] The subcategories for toxicity/adverse events were tallied among the total number of toxicity/adverse event-related discontinuations.
[6] Multiple toxicities/adverse events could have been selected.
[7] Of the two patients that had other reasons for index BTKi treatment discontinuation, one died and the other received CAR T-cell therapy.
