# Supplementary material for: Clinical Outcomes in Patients With CLL Treated With BTKi at a Large US Cancer Center
Source: Adv Hematol. 2025 Nov 30;2025:7492594. doi: 10.1155/ah/7492594 (PMC12665162; doi:10.1155/ah/7492594)
Supplement: Supplementary file 7 — Supporting Information 7 Supporting Table S6: Treatments following last BTKi treatment. [file AH-2025-7492594-s007.pdf]

Supplemental Table S6. Treatments following last BTKi treatment

	**Overall**<br>**N = 104**
**Patients with treatments after last BTKi n (%)**	62 (59.6)
**Venetoclax regimens**	50 (80.6)
Venetoclax monotherapy	23 (46.0)
Venetoclax+duvelisib	12 (24.0)
Venetoclax+rituximab	9 (18.0)
Venetoclax+obinutuzumab	4 (8.0)
Venetoclax+cyclophosphamide+prednisone+vincristine+doxorubicin	2 (4.0)
Venetoclax+cyclophosphamide+prednisone+vincristine+doxorubicin+rituximab	2 (4.0)
Venetoclax+other treatment	1 (2.0)
**Targeted therapy[1]**	7 (11.2)
Duvelisib monotherapy	2 (28.6)
Idelalisib monotherapy	2 (28.6)
Idelalisib combination therapy	1 (14.3)
Other targeted therapy	2 (28.6)
**Targeted therapy and monoclonal antibodies**	3 (4.8)
Rituximab+idelalisib	2 (66.7)
Ofatumumab+idelalisib	1 (33.3)
**Chemotherapy/steroids and monoclonal antibodies**	4 (6.5)
Chlorambucil+obinutuzumab	2 (50.0)
Cyclophosphamide+prednisone+vincristine+doxorubicin+rituximab	2 (50.0)
Rituximab+lenalidomide	1 (25.0)
**Monoclonal antibody monotherapy**	4 (6.5)
Alemtuzumab	2 (50.0)
Rituximab	1 (25.0)
Obinutuzumab	1 (25.0)
**AlloHCT**	9 (14.5)
**CAR T-cell therapy**	4 (6.5)
**Supportive care**	1 (1.6)
**Experimental therapy**	2 (3.2)
**Other**	6 (9.7)

Abbreviations: alloHCT: allogeneic hematopoietic cell transplant; BCL2; B-cell lymphoma 2; BTKi: Bruton's tyrosine kinase inhibitor; CAR: chimeric antigen receptor; N: sample size.

Notes:
[1] This does not include patients who receive venetoclax following last BTKi.
