# Supplementary material for: Clinical Outcomes in Patients With CLL Treated With BTKi at a Large US Cancer Center
Source: Adv Hematol. 2025 Nov 30;2025:7492594. doi: 10.1155/ah/7492594 (PMC12665162; doi:10.1155/ah/7492594)
Supplement: Supplementary file 8 — Supporting Information 8 Supporting Table S7: Summary of BTKi and BCL2i lines of therapy in double‐exposed and post‐BTKi and post‐BCL2i patients. [file AH-2025-7492594-s005.pdf]

Supplemental Table S7. Summary of BTKi and BCL2i lines of therapy in double-exposed and post-BTKi and post-BCL2i patients

	**Double-exposed N = 61**	**Post-BTKi and post-BCL2i N = 25**
**First BTKi line of therapy, n (%)**		
Known	60 (98.4)	25 (100.0)
1	12 (20.0)	4 (16.0)
2	19 (31.7)	5 (20.0)
3	18 (30.0)	10 (40.0)
4	10 (16.7)	5 (20.0)
≥ 5	1 (1.7)	1 (4.0)
Unknown	1 (1.6)	0 (0.0)
**First BCL2i line of therapy, n (%)**		
Known	61 (100.0)	25 (100.0)
1	0 (0.0)	0 (0.0)
2	13 (21.3)	5 (20.0)
3	12 (19.7)	4 (16.0)
4	23 (37.7)	11 (44.0)
≥ 5	13 (21.3)	5 (20.0)
Unknown	0 (0.0)	0 (0.0)
**First BTKi treatment discontinuation, n (%)**		
Still on first BTKi	0 (0.0)	0 (0.0)
Discontinued first BTKi	61 (100.0)	25 (100.0)
**Time from first BTKi treatment initiation to first BTKi treatment discontinuation, years**		
Median [Q1, Q3]	2.0 [0.8, 3.7]	2.0 [0.6, 3.5]
**First BCL2i treatment discontinuation, n (%)[1]**		
Still on first BCL2i	16 (26.2)	1 (4.0)
Discontinued first BCL2i	45 (73.8)	24 (96.0)
**Time from first BCL2i treatment initiation to first BCL2i treatment discontinuation, years[1]**		
Median [Q1, Q3]	0.8 [0.2, 2.1]	1.1 [0.4, 2.4]

Abbreviations: BCL2i: B-cell lymphoma 2 inhibitor; BTKi: Bruton's tyrosine kinase inhibitor; CAR: chimeric antigen receptor; CLL: chronic lymphocytic leukemia; mab: monoclonal antibodies; N: sample size; SD: standard deviation; SLL: small lymphocytic lymphoma; Q1: first quartile; Q3: third quartile.

Notes:

[1] Data was analyzed for the patients who discontinued their first BCL2i.
