# Supplementary material for: Clinical Outcomes in Patients With CLL Treated With BTKi at a Large US Cancer Center
Source: Adv Hematol. 2025 Nov 30;2025:7492594. doi: 10.1155/ah/7492594 (PMC12665162; doi:10.1155/ah/7492594)
Supplement: Supplementary file 9 — Supporting Information 9 Supporting Table S8: Progression‐free survival and overall survival correlation analysis. [file AH-2025-7492594-s004.pdf]

Supplemental Table S8. Progression-free survival and overall survival correlation analysis

	**Overall**<br>**N = 104**
**Patients with a valid date of assessment of response to index BTKi, n (%)[2]**	95 (90.5)
**Time from index date to progression, years[3,4]**	
Patients who progressed, n (%)	48 (50.5)
K-M, years, median (95% CI)	3.7 (2.6, 4.1)
**Overall survival, years[5,6]**	
Patients who died, n (%)	33 (35.1)
K-M, years, median (95% CI)	8.9 (6.7, NR)
**Progression-free survival, years[7]**	
Patients who progressed or died, n (%)	55 (57.9)
K-M, years, median (95% CI)	3.2 (2.6, 3.9)
**Correlation between progression-free survival and overall survival[8]**	
Correlation (95% CI)	0.79 (0.63, 0.94)

Abbreviations: BTKi: Bruton's tyrosine kinase inhibitor; CI: confidence interval; K-M: Kaplan-Meier estimator; N: sample size.

Notes:
[1] Two patients with unknown index BTKi line of therapy were excluded from the analyses by line of therapy.

[2] Patients were excluded from the analysis if the date of the clinician's assessment of response to index BTKi was missing or if treatment response was assessed after treatment discontinuation.
[3] The index date was defined as the initiation of the first treatment with a BTKi.
[4] Patients who did not progress were censored at treatment discontinuation.
[5] Overall survival was defined as time in years from index date to death from any cause. Patients who did not die during the post-index period were censored at the date of last recorded follow-up.
[6] One patient who did not die was excluded from this analysis because they did not have a date of end of follow-up.
[7] Progression-free survival was defined as the time in years from initiation of therapy to disease progression or death from any cause, whichever occurs first. Patients who did not progress nor die were censored at treatment discontinuation.
[8] Correlation between progression-free survival and overall survival was assessed using Fleischer's Pearson's correlation method of moment estimator.
